# Primary malignant melanoma of the female urethra A rare histopathology case report

**DOI:** 10.1016/j.eucr.2023.102642

**Published:** 2023-12-27

**Authors:** Anahita Ansari Djafari, Babak Javanmard, Sina Samenezhad, Amir Hossein Eslami, Azadeh Rakhshan

**Affiliations:** a Shahid Beheshti University of Medical Sciences Laser Application in Medical Sciences Research Center, Urology, Tehran, Tehran, Iran; b Department of Pathology, Shohada-e-tajrish Educational Hospital, School of Medicine, Shahid Beheshti University of Medical Sciences, Tehran, Iran

## Abstract

Malignant melanoma in the urethra is a rare tumor that is difficult to diagnose and treat, leading to a poor prognosis. In this paper, we present the case of a 36-year-old woman with history of invasive rectal adenocarcinoma (PT2N0Mx) who was tumor free for 5 years presented to urology outpatient with history of poor stream, dysuria, and dyspareuria. On examination, there was a huge mass in the meatus of urethra. Urethral malignant melanoma shows a high rate of local recurrence, about 60 % in 1 year. Overall survival in a series of 11 cases at 3 years was 27 %.

## 1 Introduction

Within all the malignancies almost 1.2 % of them are malignant melanoma, genitourinary melanoma are even rare. primary malignant melanoma is exceptionally Rare (equivocally 1 of 400000 cases of melanoma).^1^ Urethral melanomas are frequently misdiagnosed clinically, which leads to a delayed diagnosisand patients, despite all the innovation and advanced medical measures malignant melanoma of the urethra has a very poor prognosis.^2^ In this report, we present a case of melanoma originating from the urethra with history of cured invasive rectal adenocarcinoma and we discussed with patient for further options of treatment.

## 2 Case report

**Fig. 1:**
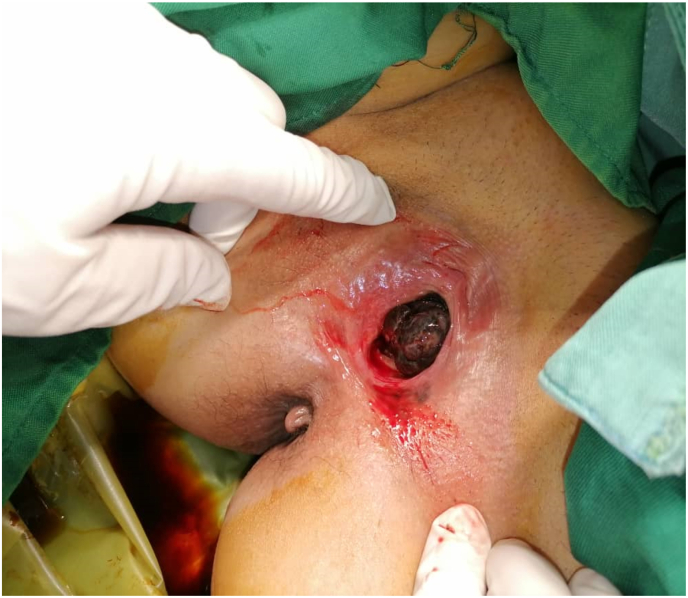
Urethral mass.

A 36 years old Female with history of invasive rectal adenocarcinoma (PT2N0Mx) who was cured by neoadjvant chemoradiotherapy (long-course radiotherapy and 5-fluorouracil) and ultra low resection (LAR). The patient who was tumor free for 5 years presented to urology outpatient with history of poor stream, dysuria, and dyspareuria And feeling of mass in meatus,Patient had this huge, firm, Necrotic, pedanculated and dark pigmented mass since 6 months ago and was diagnosed by traditional medicine doctor as genital wart or caruncle. This mass placed in the meatus of urethra without bleeding, hanging from superior wall of the urethral meatus blocking urethral entry (Fig. 1) the patient had moderate brown skin, she was passive smoker and HIV negative. There was a suspicious history of exposure to industrial paint.

**Fig. 2:**
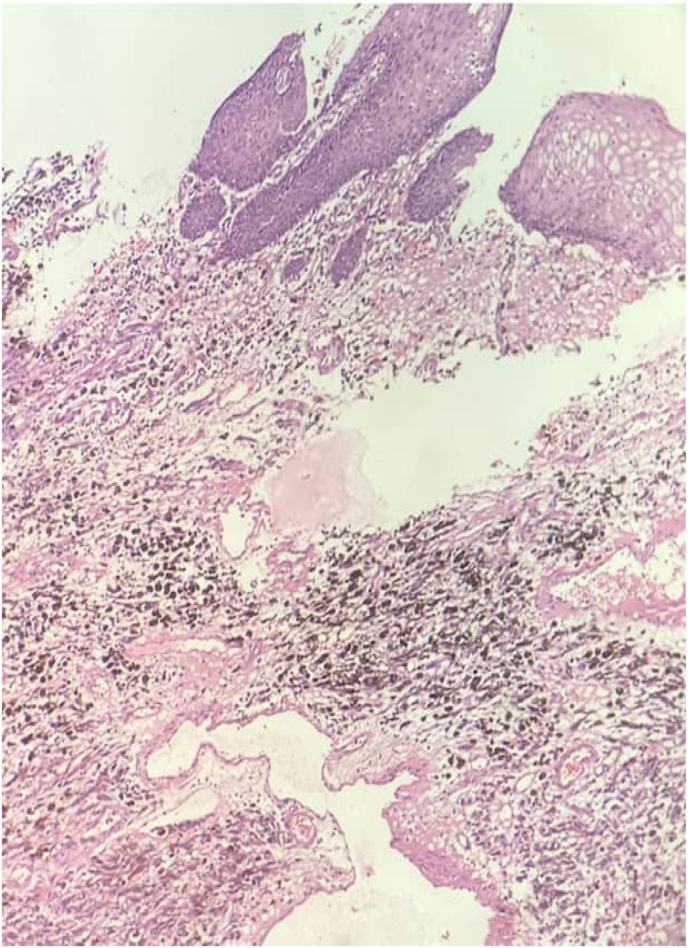
Malignant melanoma with infiltrative border and prominent intracytoplasmic melanin pigment involving the distal part of urethra which is covered by squamous epithelium (top of image).

**Fig. 3:**
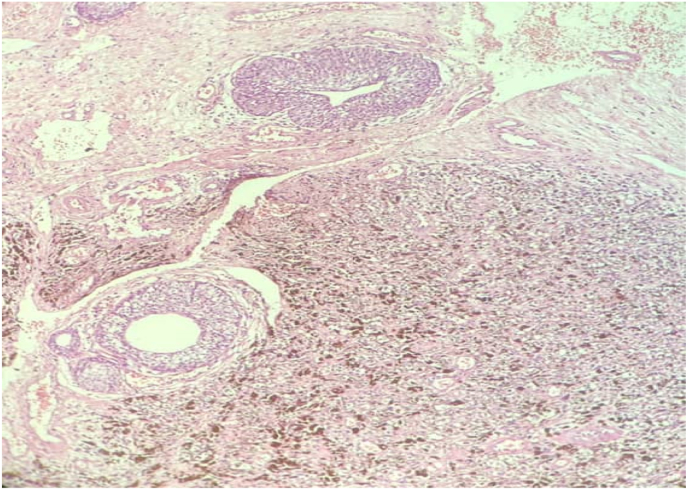
The neoplasm infiltrate among von Brunn's nests in the lamina propria (H&E stain, x40).

**Fig. 4:**
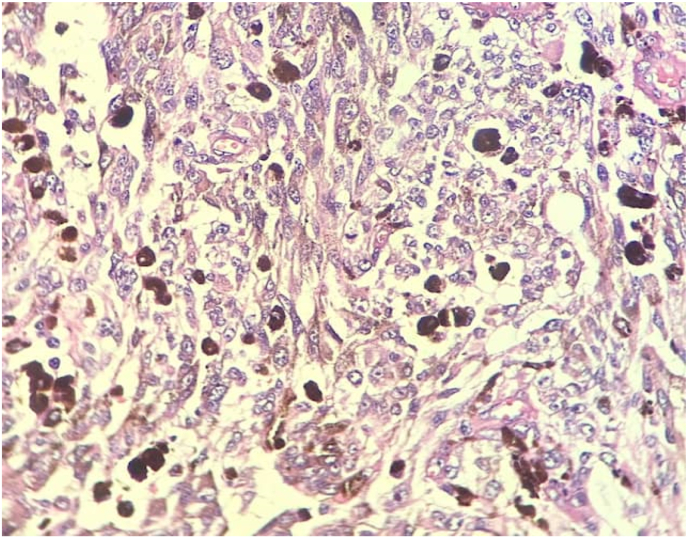
The neoplastic cells are polygonal to spindle shaped with vesicular nuclei, occasional prominent nucleoli and mitotic activity. Conspicuous melanin pigment in the cytoplasm of tumoral cells is seen (H&E stain, x400).

**Fig. 5:**
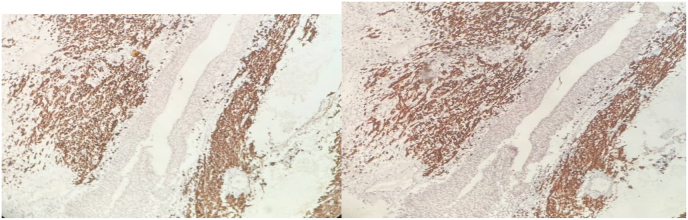
The diagnosis of melanoma is confirmed by IHC study for HMB-45 and Melan-A markers respectively which show diffuse cytoplasmic immunoreactivity (immunohistochemical staining, x40).

On general examination, no skindiscoloration or abnormality including any cutaneous lesions was found. No organomegaly or lump was found on an abdominal examination. There is no evidence of metastasis in Chest and abdominopelvic CT Scan. In cystoscopy there is no fistula and dye test was normal, the mass was resected by wide local resection under Spinal anesthesia without any further actions, after removing foley catheter patient was able to urinate and had no complaints about incontinency. The pathology report of the mass as follow (Fig. 2, Fig. 3, Fig. 4, Fig. 5):

Pathology report confirmed free margin resection, given the fact that urethral melanoma is a rare condition they suggested advanced staining to confirm the diagnosis.

## 3 Discussion

Within all the malignancies almost 1.2 % of them are malignant melanoma, genitourinary melanoma are even rare. primary malignant melanoma is exceptionally Rare (equivocally 1 of 400000 cases of melanoma).^1^

In 1896, The very first documented case of urethral melanoma was reported by tyrell and reed, thereafter in last 60 years only 121 cases of malignant melanoma was inscribed.^3^

The mostly known presenting symptoms of urethral melanoma are: dysuria with decreased force and caliber of urinary flow, sensation of mass, vaginal bleeding and haematuria. Females are 3 times more likely to have urethral melanoma with the average age of 68 years^4^^,^^5^

There is a very broad list of differential diagnosis when it comes to urethral melanoma such as benign lesions from lentiginous hyperplasia to melanocytic nevi to malignant lesions from lymphoma to sarcoma to even plasmacytoma. As our differential diagnosis expands, there is a huge packs of different patterns for pathologists to get the precise diagnosis. Main role of pathological diagnosis rely on immunohistochemical staining where the most used markers (HMB45) that is highly specific for melanocytic neoplasms confirms the diagnosis. in our case IHC study with HMB-45 showed diffuse cytoplasmic immunoreactivity confirming the diagnosis of urethral melanoma.^6^

There has been a controversy in treatment recommendations and definite management of primay malignant melanoma. It may primarily depend on site of tumor and and clinical stage as TNM classification by American joint committee on cancer (AJCC) giving us crucial edge to address the issue.

There is no united word for treatment of urethral melanomas in women but the main effective idea is surgical excision as wide as possible to get the free margin as surgeons even called for pelvic exentrations. With very low and limited number of patients and low rate of survival in urethral melanoma (almost 2 years) there is no definitive conclusions.^1^

New studies have shown a very effective and well tolerated benefits of target therapy for mucosal melanoma not only but after aggressive surgery however there was no big differences between aggressive surgery and conservative surgery. Target therapy can be done with or without immunotherapy.^7^ Urethral malignant melanoma despite all the measures has a very high chance of recurrence (almost 6 out of 10 patients in 1 year follow up).^1^

The present patient showed local recurrence in 2 months of follow up (On genital examination under anesthesia 2 sites of necrotic lesion on dome of vagina noted). After consultation the patient referred to oncologist for targeted therapy.

## Consent

Written informed consent was obtained from the patient for the publication of this case report.

## Funding

No funding was recieved for conducting this study.

## CRediT authorship contribution statement

**Anahita Ansari Djafari:** Writing – original draft. **Babak Javanmard:** Writing – original draft, Supervision. **Sina Samenezhad:** Writing – original draft, Visualization. **Amir Hossein Eslami:** Writing – review & editing. **Azadeh Rakhshan:** Writing – original draft, Investigation.

## Declaration of competing interest

The authors declare they have no financial interests.

The authors report no conflict of interest.
